# Could the Biological Potency of *Porcelia macrocarpa* R.E. Fries (Annonaceae) be Linked to Its Diverse Metabolite Profile?

**DOI:** 10.1002/cbdv.202501248

**Published:** 2025-11-14

**Authors:** Fernanda Thevenard, Ivanildo A. Brito, Emerson A. Oliveira, Mariana H. Chaves, João Henrique G. Lago

**Affiliations:** ^1^ Center For Natural and Human Sciences Federal University of ABC São Paulo Brazil; ^2^ Department of Chemistry Federal University of Piauí Teresina Brazil

**Keywords:** acetogenins, alkaloids, biological activity, flavonoids, *Porcelia macrocarpa*, secondary metabolites, terpenes

## Abstract

*Porcelia macrocarpa* is a Brazilian plant species belonging to the Annonaceae family. To date, more than 190 compounds have been identified and/or isolated from this species, with terpenoids comprising the majority (62%). Alkaloids represent the second most abundant class (11%), followed by steroids (4%), acetogenins (4%), fatty acids (4%), arylpropanoids (3%), and flavonoids (1%). These metabolites exhibit a wide range of biological activities, including antibacterial, antifungal, antiviral, cytotoxic, anti‐inflammatory, antiparasitic, and antioxidant effects. This review highlights the remarkable chemical profile of *P. macrocarpa* and the pharmacological potential of the metabolites identified in this species, offering valuable insights into its possible applications and encouraging further research across multiple scientific and medical fields.

## Graphical abstract

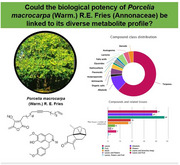

## 1 INTRODUCTION

**FIGURE 1:**
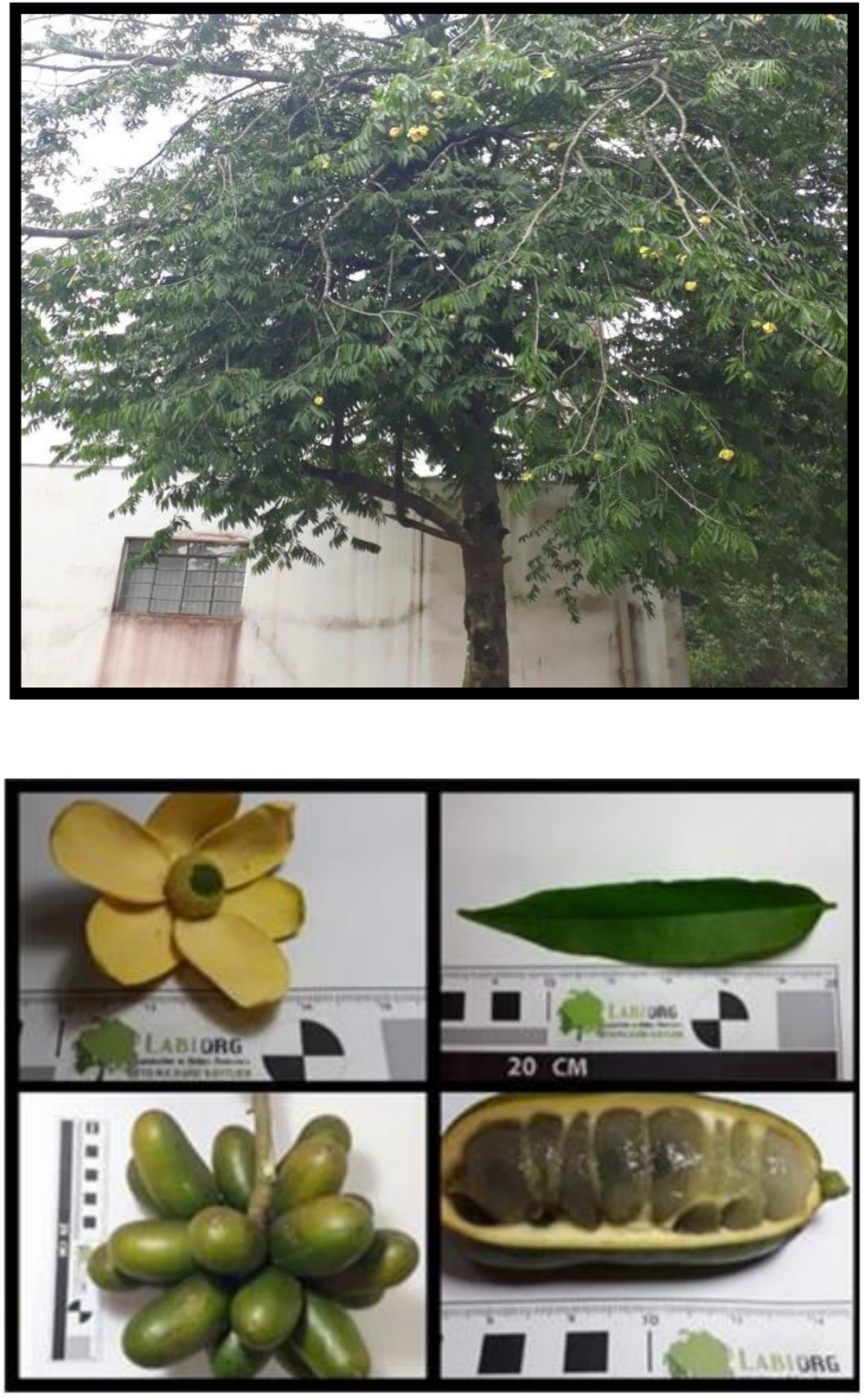
Adult tree and different parts (flowers, leaves, fruits, and seeds) of *P. macrocarpa*.

Natural products offer a wide range of compounds with biological activity and structural diversity [1, 2]. Plants, in particular, have been used for thousands of years in traditional medicine to treat different diseases [2]. Belonging to the Annonaceae family, the genus *Porcelia* comprises approximately 18 species—including *P. macrocarpa, P. magnifructa, P. magnifructum, P. steinbachii, P. venezuelensis, P. nitidifolia, P. cinnamonema, P. ponderosa, P. pondemsa, P. saffordiana, P. mediocris, P. discolor, P. grandiflora, P. parviflora, P. pygmaea, P. triloba*, and *P. stenopetala*—distributed across tropical regions of the Americas. Despite this taxonomic diversity, chemical and biological studies have been conducted exclusively on *P. macrocarpa* (Figure 1), with only one recent study addressing *P. ponderosa* [3]. Various classes of compounds—including alkaloids, flavonoids, arylpropanoids, terpenoids, acetogenins, and others—have been reported for *P. macrocarpa*, contributing to its observed biological activities such as cytotoxic, antibacterial, antifungal, anti‐inflammatory, antioxidant, and antiparasitic effects. Given the genus's potential in the search for novel bioactive compounds, this review provides an overview of substances isolated from *P. macrocarpa* and their activity in different biological models.

## 2 Chemical Composition and Biological Potential of *Porcelia macrocarpa*

**FIGURE 2:**
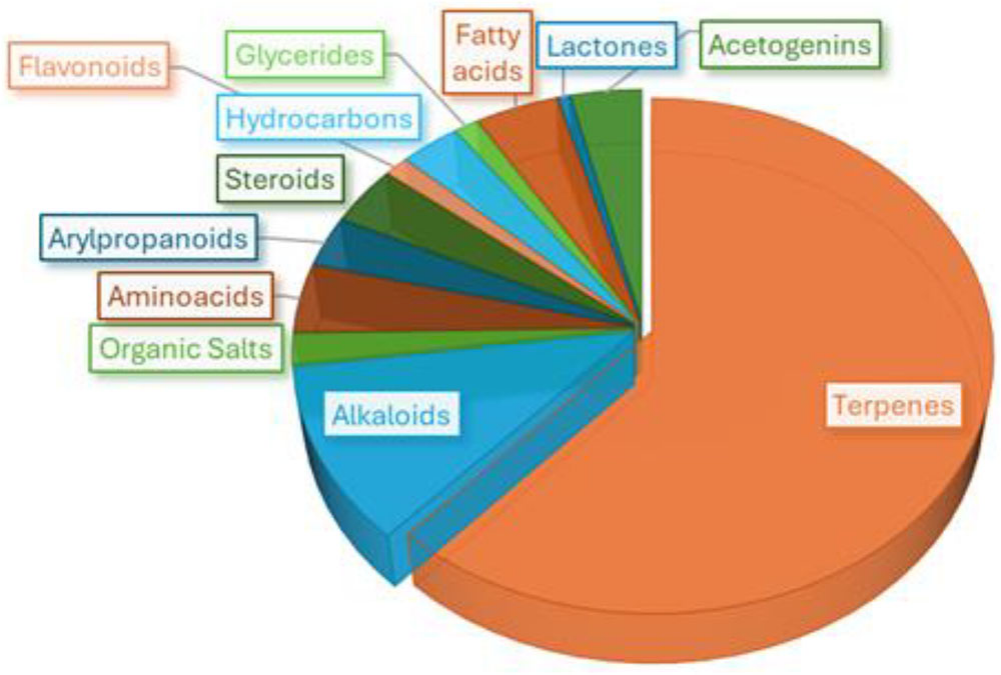
Distribution of metabolites found in *P. macrocarpa*.

*P. macrocarpa* has been the subject of phytochemical investigations by various Brazilian researchers. To date, a total of 193 compounds have been reported from this species. Terpenes represent the predominant class (62%), reflecting the nature of the plant tissues analyzed. Alkaloids constitute the second most abundant group (11%), while other classes such as flavonoids (1%), amino acids (4%), organic salts (2%), arylpropanoids (3%), steroids (4%), lactones (1%), acetogenins (4%), fatty acids (4%), hydrocarbons (3%), and glycerides (1%) are found in smaller proportions. A visual summary of this distribution is presented in Figure 2.

**FIGURE 3:**
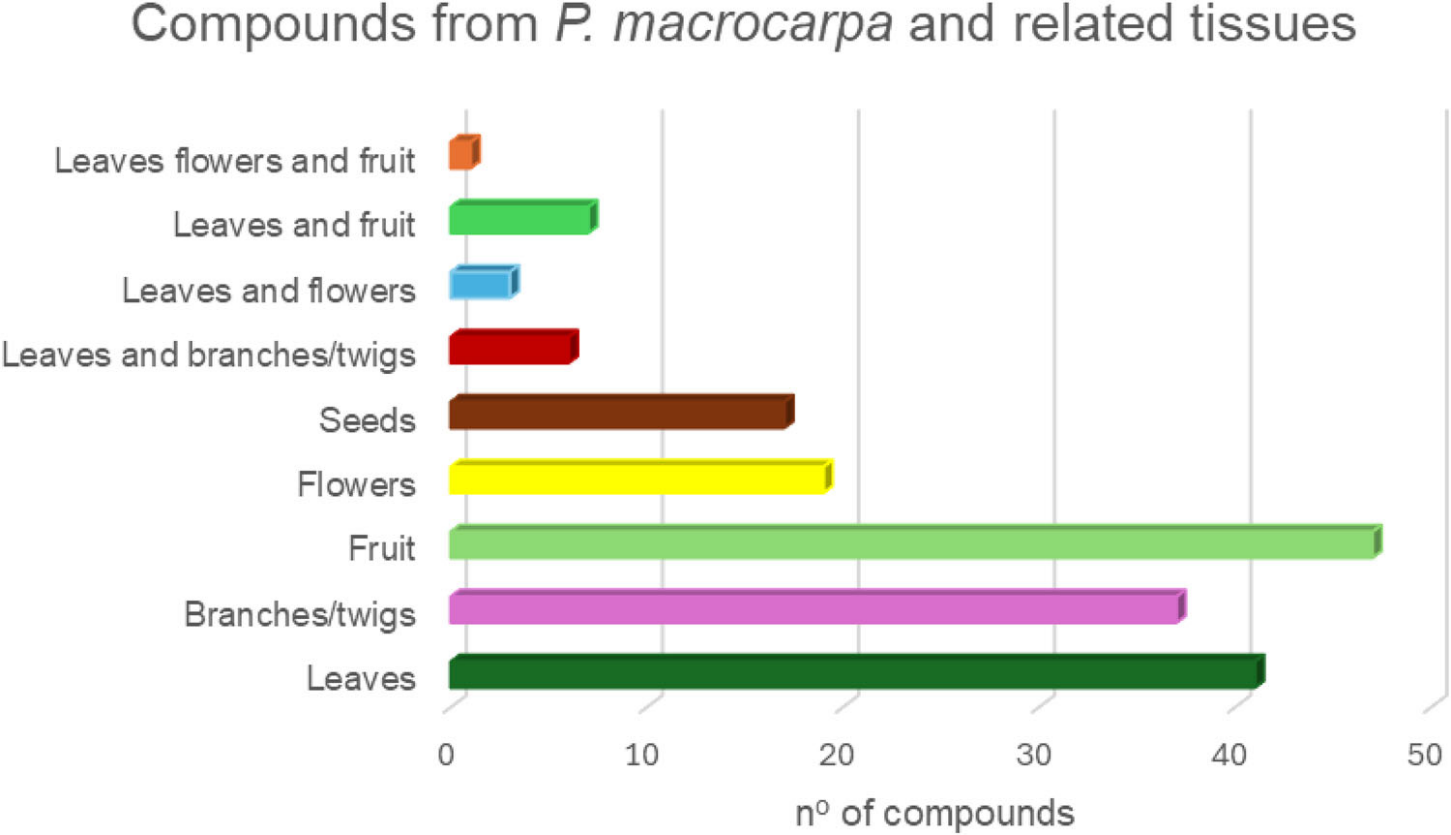
Number of compounds related in different tissues of *P. macrocarpa*.

Among the plant tissues investigated to date, the majority of compounds have been identified and/or isolated from the fruits of *P. macrocarpa*, followed by branches/twigs and leaves. Figure 3 illustrates the distribution of compounds according to their tissue of origin.

### 2.1 Terpenoids

A total of 100 terpenoids have been identified and/or isolated from *P. macrocarpa*, and in this review, they are categorized into subclasses: mono‐, sesqui‐, di‐, and hemiterpenes. Among them, 21 monoterpenes (compounds **1–21**) were identified in the essential oils from fruits and leaves [4, 5, 6]. The essential oils extracted from the leaves of *P. macrocarpa* were constituted basically by a mixture of sesquiterpenes; however, one monoterpene (verbanyl acetate ‐ **21**) and one diterpene (phytol – **94**) were also identified. In contrast, the essential oil from the fruits consisted of 65 individual components, with monoterpenes representing the predominant class (45%). Among these, neryl formate (**11**) and geranyl formate (**12**) were the most abundant, accounting for 8.8% and 27.3% of the oil composition, respectively. The leaf essential oil also exhibited notable antifungal activity, with minimum inhibitory concentration (MIC) values of 0.50 and 0.06 µg/mL against *Cryptococcus neoformans* serotypes A and D, respectively, and 1.00 µg/mL against *Cryptococcus gattii* serotypes [4]. Isolated α‐pinene (**16**) demonstrated antinociceptive activity in the mouse tail‐flick test [7], while pure camphene (**17**) exhibited insecticidal activity, with an LC_50_ of 10.64 µg/mL against *Helicoverpa armigera* larvae and a 50% effective concentration (EC_50_) of 35.39 µg/mL against its eggs [8].

The majority of metabolites identified from *P. macrocarpa* are sesquiterpenes, accounting for 76% of the total, with 72 compounds (**22–93**) primarily found in the essential oils. Among them, germacrene D (**23**) and bicyclogermacrene (**24**) were the predominant constituents in the leaves, representing 28.8% and 23.9% of the composition, respectively. Additionally, sesquiterpenes comprised 37% of the essential oil composition in ripe fruits of *P. macrocarpa* [4]. Pure δ‐cadinene (**36**) exhibited antibacterial activity against *Listeria monocytogenes*, with a MIC of 5 µg/mL and an MBC of 10 µg/mL. It also inhibited biofilm formation, altered cell morphology, and caused damage to the bacterial cell membrane [9]. Humulene (**59**) displayed an LC_50_ of 20.86 µg/mL against *H. armigera* larvae and an EC_50_ of 77.10 µg/mL against *H. armigera* eggs [8]. Additionally, humulene (**59**) has demonstrated anticancer activity against various cell lines both in vitro and in vivo [10]. Cyperotundone (**88**), in combination with adriamycin, significantly inhibited the growth of MCF‐7 breast cancer cells by inducing cell cycle arrest and apoptosis, without causing significant toxicity at a dose of 100 mg/kg/day over 15 days [11].

Six diterpenes—*E*‐phytol (**94**), isophytol (**95**), 3*Z*‐cembrane A (**96**), *E,E*‐geranyl linalool (**97**), manool (**98**), and *E*‐phytol acetate (**99**) have been isolated from the fruit or flowers of *P. macrocarpa* [4, 12, 13]. Previous studies [14] reported that pure diterpene **94** demonstrated suppression of P‐glycoprotein (P‐gp) activity in an *in vitro* multidrug‐resistant acute myeloid leukemia model treated with doxorubicin, suggesting its potential role as a non‐toxic modulator of this protein.

The hemiterpene 3‐methylenebutane‐1,2,4‐triol (**100**) was identified through chromatographic separation and analysis of the aqueous fraction of *P. macrocarpa* branches [12, 15]. However, its biological activity has not yet been reported.

### 2.2 Alkaloids

Several alkaloids have been isolated from leaves and/or branches/twigs of *P. macrocarpa* including four bisbenzylisoquinolines: reticuline (**101**), 4’‐methylcoclaurine (**102**), coclaurine (**103**), and norjuziphine (**104**), four aprorphines: asimilobine (**105**), michelalbine (**106**), stefarine (**107**), and liriodenine (**108**), five azaanthraquinones: cleistopholine (**109**), 6‐methoxycleistopholine (**110**), 6,7‐dimethoxycleistopholine (**111**), 5‐hydroxy‐6‐methoxycleistopholine (**112**), 5‐hydroxy‐6,7‐dimethoxycleistopholine (**113**), and four azafluorenones: onichine (**114**), 6‐methoxyonichine (**115**), 6,7‐ dimethoxyonichine (**116**), and 7‐methoxyonichine (**117**). Additionally, 2‐methoxy‐3‐(1‐methylpropyl)‐pyrazine (**118**) was identified in its flowers [12, 16]. The alkaloid **102**, isolated from branches, was tested against several fungal species, showing a MIC of 25 µg/mL against *Cladosporium cladosporioides* and *Cladosporium sphaerospermum* [17]. Reticuline (**101**) exhibited activity against *Staphylococcus epidermidis* (ATCC 12228 and 6ep) as well as *Candida albicans* and *Candida parapsilosis*, with a MIC of 100 µg/mL [18]. Additionally, neurotoxic effects have also been associated with reticuline (**101**) [19]. Alkaloid **102** has also demonstrated anticancer activity against human lung adenocarcinoma and colon carcinoma cell lines, with an ED_50_ greater than 50 µg/mL, along with additional cytotoxic effects [20].

Alkaloid **104** was evaluated for anti‐platelet aggregation, showing complete or near complete inhibition of aggregation induced by thrombin, arachidonic acid, collagen, and platelet aggregating factor at 100 µg/mL [21]. Coclaurine (**103**) has demonstrated strong anti‐HIV activity, with an EC_50_ of 0.8 µg/mL and a therapeutic index greater than 125. It has also exhibited anticancer effects, showing IC_50_ values of 8.2 µg/mL against HCT116 colon cancer cells, 15.3 µg/mL against MCF‐7 human breast cancer cells, and 1.7 µg/mL against HepG2 human liver cancer cells. For comparison, the standard chemotherapeutic agent doxorubicin displayed IC_50_ values of 0.8, 1.3, and 0.8 µg/mL, respectively [22, 23]. In addition, alkaloid **103** has shown in vitro antispasmodic activity, with an IC_50_ of 14.3 µM of the tonic phase of the vas deferens response to potassium [24].

Asimilobine (**105**) exhibited notable antioxidant activity and demonstrated antimicrobial effects against *S. epidermidis* (6ep), the reference strain *Kocuria rhizophila* (ATCC 9341), and *Candida dubliniensis* (ATCC 777 and ATCC 778157), with a MIC of 50 µg/mL for all three microorganisms [18]. Antiproliferative effects were observed against AGS and DU‐145 cell lines with IC_50_> 500 µM [19]. Antidepressant effects have also been reported for asimilobine [20]. Similarly, liriodenine (**108**) showed antimicrobial activity against *S. epidermidis* (6ep) and *C. dubliensis* (ATCC 777) with an MIC of 50 µg/mL [18]. Anticancer activity against various cell lines has also been reported for liriodenine [19]. Additionally, liriodenine was evaluated against coronavirus disease and showed inhibitory activity of 24.8% [25].

Cleistopholine (**109**), a mixture of 6‐methyoxycleistofoline (**110**) and 6,7‐dimethoxycleistopholine (**111**), as well as pure 5‐hydroxy‐6,7‐dimethyoxycleistopholine (**113**), were evaluated against *C. cladosporioides* and *C. sphaerospermum*. Compound **109** showed MIC values of 1.0 µg against both fungi. The mixture of **110** and **111** also showed antifungal activity with MICs of 5.0 and 1.0 µg against *C. cladosporioides* and *C. sphaerospermum*, respectively. The mixture of **110** and **111** showed DNA‐damaging effects, which was also observed for pure **113** [17]. Alkaloid **109** also showed some antimicrobial activity against *S. epidermidis* (ATCC 12228 and 6ep), *Enterococcus faecalis* (Ef), and *C. dubliniensis* (ATCC 778157) with an MIC of 250 µg/mL [18] and exhibited potent cytotoxicity against Hep G2 (human hepatoma cell) and Hep 2,2,15 (Hep G2 transfected with hepatitis B) cell lines with IC_50_ values of 0.22 µg/mL and 0.54 µg/mL, respectively [19].

Alkaloid **113** exhibited antifungal activity against *C. cladosporioides* and *C. sphaerospermum*, inhibiting fungal growth at concentrations of 1.0 µg and 5.0 µg, respectively. Alkaloids **114–116** also demonstrated antifungal effects. The minimum inhibitory amount of onichine (**114**) required for *C. cladosporioides* and *C. sphaerospermum* was 1.0 µg, while the mixture of compounds **115** and **116** inhibited these fungi at 5.0 µg and 1.0 µg, respectively. Additionally, 7‐methoxyonichine (**117**) showed DNA‐modifying potential against the yeast strain RS 321, with an IC_12_ of 47 µg/mL, a similar effect observed for the **115+116** mixture [17].

### 2.3 Amino Acids

Amino acids citrulline (**119**), pipecolic acid (**120**), L‐valine (**121**), L‐proline (**122**), L‐alanine (**123**), γ‐aminobutyric acid (**124**), and 4‐hydroxyproline (**125**) were isolated from *P. macrocarpa* branches [4, 12]. To the best of our knowledge, no biological activities have been reported for these compounds to date.

### 2.4 Arylpropanoids

Five arylpropanoids were isolated from *P. macrocarpa* branches: *N‐trans*‐feruloyltyramine (**126**), *N‐trans*‐cafeoyltyramine (**127**), *N‐trans*‐sinapoyltyramine (**128**), and lignanamides A (**129**) and B (**130**) [12, 15, 26]. Previous studies reported that compound **126** showed higher anti‐diabetic potential than the standard drug acarbose by inhibiting α‐glucosidase activity in vitro, with an IC_50_ value of 3.58 µM [27], and displayed anti‐inflammatory/neuroinflammatory properties [28, 29]. Compounds **126** and **127** were found to influence human gut microbiota in vitro [30] and demonstrated antifungal activity against *Botrytis cinerea* at a minimum concentration of 10 ppm. Notably, only compound **126** inhibited *Aspergillus niger* and *Penicillium italicum* at the same concentration [31]. In addition, compounds **126** and **127** showed antibacterial activity against *Escherichia coli* (MIC of 15.7 and 31.3 µg/mL, respectively), *Pneumonia aeruginosa* (MIC of 15.7 and 62.5 µg/mL, respectively), *Staphylococcus epidermis* (MIC of 15.7 and 31.3 µg/mL, respectively), and *Staphylococcus aureus* (MIC of 7.8 and 15.7 µg/mL, respectively). Furthermore, compounds **126** and **127** displayed cytotoxicity against Hep3B and MCF cells (IC_50_ values between 4 and 15 µM) [32]. Compounds **126** and **128** showed in vitro cytotoxic activity against cancerous HeLa cells, with IC_50_ values of 19.95 and 9.77 µM, respectively [33, 34]. However, no biological effects were reported for lignanamides **129** and **130** [51, 52].

### 2.5 Steroids

Mixtures of free and glycosylated steroids stimast‐4‐en‐3‐one (sitostenone – **131**), 3β‐stigmata‐4,22,25‐trien‐3‐ol (**132**), stigmasta‐4,25‐dien‐3‐one (**133**), stigmasta‐4,22*E*,25‐trien‐3‐one (**134**), 3‐O‐β‐glucosyl‐sistosta‐5,25‐dien‐3‐ol (**135**), and 3‐O‐β‐glucosyl‐sistosta‐5,22,25‐trien‐3‐ol (**136**) were obtained from *P. macrocarpa* branches [12, 35]. To the best of our knowledge, no biological activity has been reported for these steroids.

### 2.6 Flavonoids

Flavonoids 3‐*O*‐rutinosyl‐7,4'‐O‐dimethyl quercetin (**137**) and 3‐*O*‐rutinosyl‐5β‐*O*‐glucopyranosyl‐7,4'‐*O*‐dimethyl quercetin (**138**) were isolated from the branches of *P. macrocarpa* [12, 15, 35]. To the best of our knowledge, no biological activity has been reported for these compounds.

### 2.7 Hydrocarbons

Four hydrocarbons have been identified in the essential oils of *P. macrocarpa*: 1‐octadecene (**139**), pentacosane (**140**), hexacosane (**141**), and heptacosane (**142**) [4]. Additionally, toluene (**143**) has been identified in the flower of this plant [5]. As reported in the literature [14], compound **142** has been identified as both a substrate and inhibitor of P‐gp, enhancing the intracellular retention of doxorubicin. This property suggests its potential in overcoming drug resistance in the treatment of acute myeloid leukemia.

### 2.8 Organic Salts

Three organic salts were isolated and identified from the branches of *P. macrocarpa*: tyrosine trimethylammonium (**144**), glycine trimethylammonium (**145**), and choline (**146**) chlorides [12, 15]. To the best of our knowledge, no biological activity has been reported for these compounds.

### 2.9 Acylglycerols

Two acylglycerol derivatives, α,α’‐dimacrocarpoyl‐β‐oleylglycerol (**147**) and α‐macrocarpoyl‐α’‐oleylglycerol (**148**) were isolated from seeds of *P. macrocarpa* [36]. To the best of our knowledge, no biological activity has been reported for these compounds.

### 2.10 Fatty Acids

Several natural products of the polyketide class, especially the fatty acids eicos‐11‐yn‐19‐enoic acid (**149**), 12,14‐octadecadiynoic acid (macrocarpic acid – **150**), octadec‐11‐en‐9‐ynoic acid (santalbic acid – **151**), 8‐hydroxyoctadeca‐9,11‐diynoic acid (**152**), 8‐hydroxyoctadec‐17‐ene‐9,11‐diynoic acid (**153**), octadec‐9‐ynoic acid (stearolic acid ‐ **154**), and docos‐13‐yn‐21‐enoic acid (**155**) were isolated from seeds of *P. macrocarpa*. Fatty acids **150–152** and **154** showed anti‐*T. cruzi* activity with EC_50_ values of 38.7, 59.9, 57.3, and 27.6 µM against the trypomastigote forms of the parasite, respectively [37]. Fatty acid **155** showed antileishmanial activity against amastigotes of *Leishmania infantum* with an EC_50_ of 48.5 µM [38].

### 2.11 Lactone

The lactone γ‐dodelactone (**156**) has been identified in the essential oil of *P. macrocarpa* fruits [4]. To the best of our knowledge, no biological activity has been reported for this compound.

### 2.12 Acetogenins

Sixteen acetylene acetogenins were isolated from seeds of *P. macrocarpa* and were identified as 3‐hydroxy‐4‐methyl‐2‐(*n*‐eicos‐11’‐yn‐19’‐enyl)butanolide (**157**), 3‐hydroxy‐4‐methyl‐2‐(*n*‐eicos‐11’‐ynyl)butanolide (**158**), 3‐hydroxy‐4‐methylen‐2‐(*n*‐eicos‐11′‐yn‐19′‐enyl)but‐2‐enolide (**159**), (4*R*)‐3‐hydroxy‐4‐methyl‐2‐(*n*‐eicos‐11′‐yn‐19′‐enyl)but‐2‐enolide (**160**), 3‐hydroxy‐4‐metilen‐2‐(octadeca‐9’‐yn‐17’‐enyl)but‐2‐enolide (**161**), 3‐hydroxy‐4‐methylen‐2‐(*n*‐hexadeca‐7’‐yn‐15’‐enyl)but‐2‐enolide (**162**), 3‐hydroxy‐4‐methyl‐2‐(*n*‐octadec‐11′‐yn‐17′‐enyl)butanolide (**163**), 3‐hydroxy‐4‐methyl‐2‐(*n*‐docos‐11′‐yn‐21′‐enyl)butanolide (**164**), 3‐hydroxy‐4‐methyl‐2‐(*n*‐tetracos‐11′‐yn‐23′‐enyl)butanolide (**165**), 3‐hydroxy‐4‐methyl‐2‐(*n*‐octadec‐11′‐ynyl)butanolide (**166**), 3‐hydroxy‐4‐methyl‐2‐(*n*‐docos‐11′‐ynyl)butanolide (**167**), 3‐hydroxy‐4‐methyl‐2‐(*n*‐tetracos‐11′‐ynyl)butanolide (**168**), 3‐hydroxy4‐methyl‐2‐(*n*‐octadeca‐13′,17′‐dien‐11′‐ynyl)butanolide (**169**), 3‐hydroxy‐4‐methyl‐2‐(*n*‐eicosa‐13′,19′‐dien‐11′‐ynyl)butanolide (**170**), 3‐hydroxy‐4‐methyl‐2‐(*n*‐octadec‐13′‐en11′‐ynyl)butanolide (**171**), and 3‐hydroxy‐4‐methyl‐2‐(*n*‐eicosa‐13′‐en‐11′‐ynyl)butanolide (**172**). Following stereochemical analysis, including vibrational circular dichroism, the absolute configuration of the γ‐lactone moiety in acetogenins **157**, **158**, and **163–172** was determined to be (2*S*, 3*R*, 4*R*). Acetogenins **157**, **159**, and **160** exhibited in vitro activity against *Trypanosoma cruzi*, with EC_50_ values of 7.8, 0.4, and 3.6 µM, respectively, against trypomastigotes, and 58.3, 23.0, and 27.7 µM against amastigotes. In comparison, the reference drug benznidazole showed EC_50_ values of 18.7 µM for trypomastigotes and 5.5 µM for amastigotes. Notably, no cytotoxicity was detected in mammalian cells at the highest concentration tested (CC_50_> 200 µM or 200 µg/mL) for any of the acetogenins, except compound **159**, which exhibited a CC_50_ of 80 µM against NCTC cells [39]. Compounds **157** and **160–162** exhibited antileishmanial activity against the amastigote forms of *L*. *infantum*, with EC_50_ values of 29.9, 9.2, 10.4, and 11.0 µM, respectively. While compound **157** showed no cytotoxicity in this assay, compounds **160–162** presented CC_50_ values of 80.0, 82.9, and 85.4 µM [38]. Compound **158** alone showed no activity against *L. infantum*; however, mixtures of **157** and **158** in various proportions displayed significant activity, suggesting a potential synergistic effect [40].

The homologous series of acetogenins **157**, **158**, and **163–168** was evaluated against *Trypanosoma cruzi* amastigotes. Compounds **157**, **158**, **163**, **164**, **167**, and **168** exhibited EC_50_ values ranging from 13.9 to 1.1 µM, with compound **168** being the most potent. Notably, compound **168** also showed the highest selectivity index (SI > 181.8), outperforming the reference drug benznidazole (SI > 55.5). In contrast, compounds **165** and **166** were inactive (EC_50_> 100 µM). The findings highlight that side‐chain flexibility and elongation significantly influence anti‐*T. cruzi* activity. No cytotoxicity against mammalian cells was observed for any of the compounds tested [41].

A mixture of enyne acetogenins **169–172** was evaluated against *T. cruzi*, showing EC_50_ values of 4.9 µg/mL for trypomastigotes and 2.5 µg/mL for amastigotes—comparable to the standard drug benznidazole, which exhibited EC_50_ values of 4.8 and 1.4 µg/mL, respectively. The SI of the mixture against amastigotes was more than twice that of benznidazole, with an SI > 83. No cytotoxicity was observed at the highest concentration tested [42].

### 2.13 Other Compounds

Other compounds (**173–193**) have also been reported in *P. macrocarpa* [4, 5, 12, 15, 26, 43]. Phenylacetaldehyde (**176**) showed uncompetitive reversible inhibition of fungal tyrosinase with an IC_50_ of 0.39 µM, more effective than the standard arbutin (IC_50_ = 30 µM) [44]. Methyl salicylate (**177**) showed low acaricidal potential, with a mean mortality of 0.58% of *Boophilus microplus* larvae at 1% concentration [45]. Ethyl pentanoate (**186**), at 0.01 MIC, stimulated the production of a novel extracellular protein by *Bacillus subtilis*. This protein exhibited quorum‐sensing inhibition—an effect that can reduce pathogenicity—resulting in inhibition zones ranging from 10.00 to 10.33 mm [46]. Liriodendrin (**193**) showed in vivo anti‐asthmatic activity in mice, improving airway hyperresponsiveness at 15 mg/kg [47]. Another study also evaluated the anti‐inflammatory and antinociceptive effects of this compound *in vivo*, showing that liriodendrin (administered at 5 and 10 mg/kg/day) significantly reduced vascular permeability and alleviated acute paw edema [48].

All compound structures are shown in Figure 4, while Table 1 lists the *P. macrocarpa* tissue in which they were identified and the associated biological activity.

FIGURE 4Structures of compounds **1**–**193**, from *Porcelia macrocarpa*.

**Figure:**
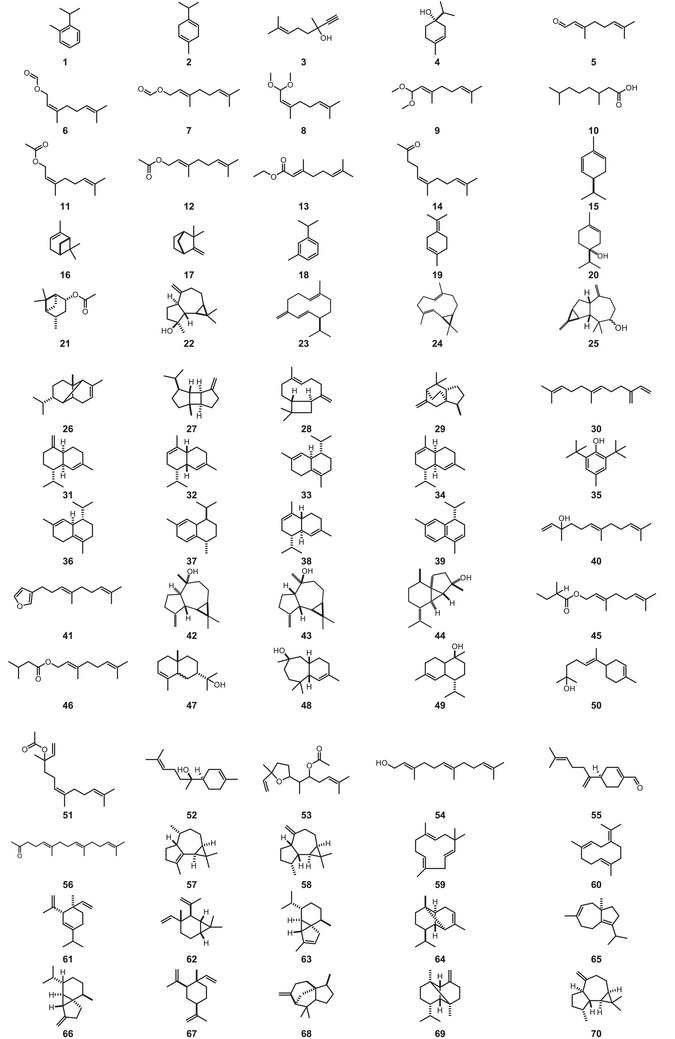

**Figure:**
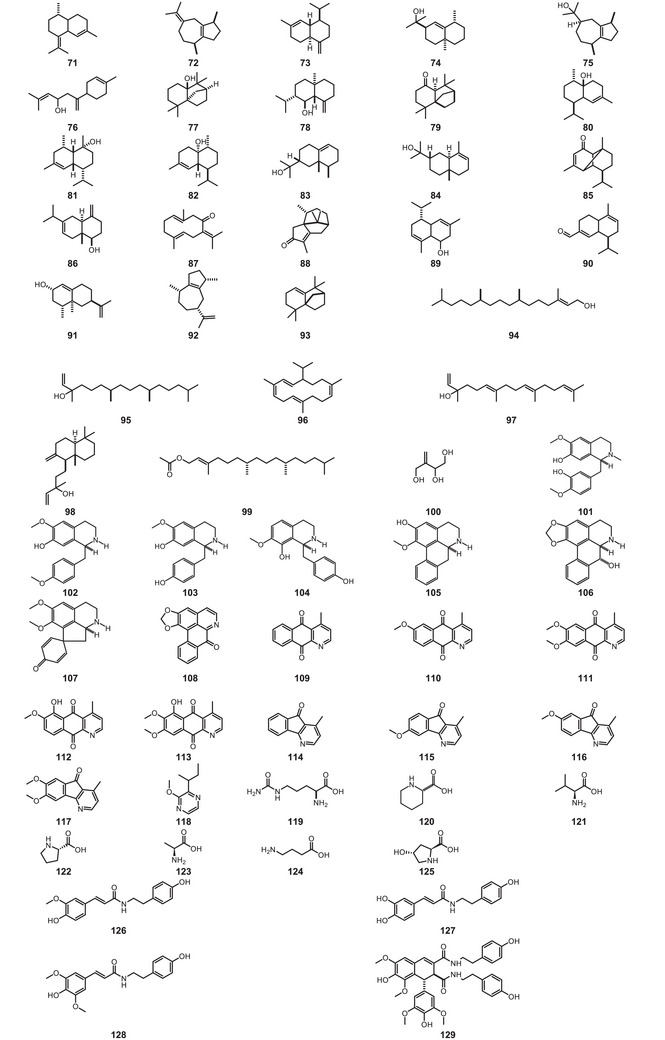

**Figure:**
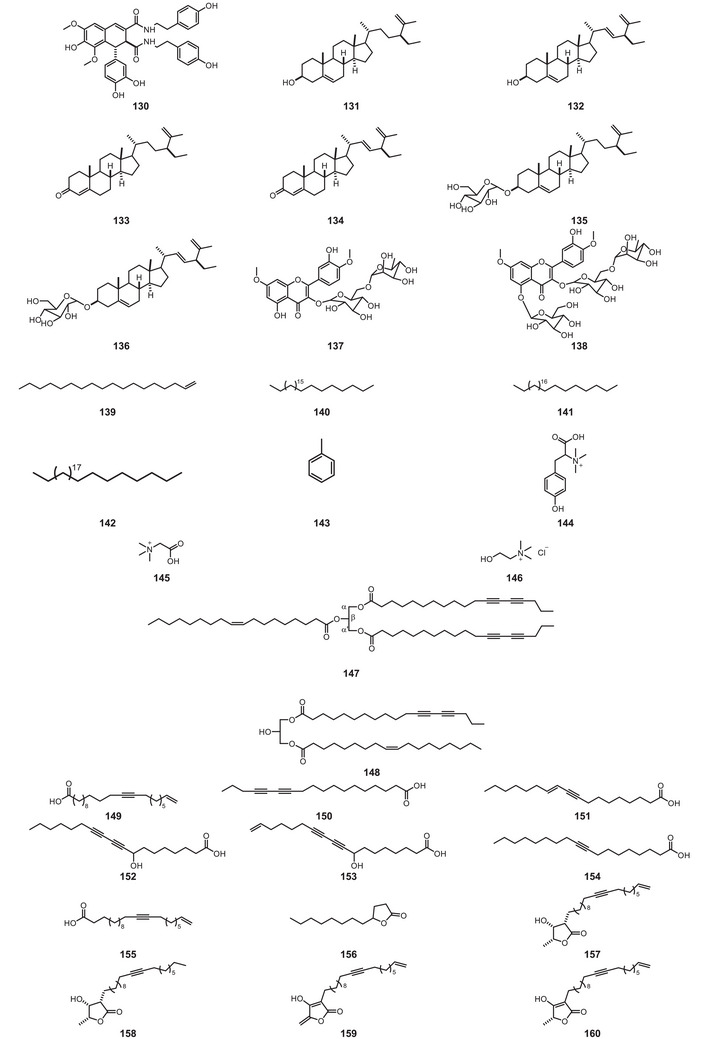

**Figure:**
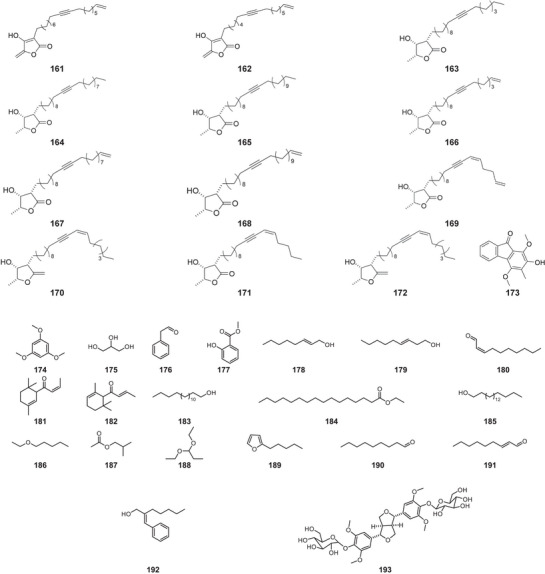

**TABLE 1:** Compounds from *Porcelia macrocarpa* separated by class, plant tissue, and biological activity.

*Plant tissue*	*Compounds*		*Biological activity*	*Reference*
**Monoterpenes**				
Fruits	*o*‐cymene	**1**	—	49
Fruits	γ‐terpinene	**2**	—	49
Fruits	dehydrolinalool	**3**	—	49
Fruits	terpinen‐4β‐ol	**4**	—	49
Fruits	geranial	**5**	—	49
Fruits	neryl formate	**6**	—	49
Fruits	geranyl formate	**7**	—	49
Fruits	dimethoxy‐*Z*‐citral	**8**	—	49
Fruits	dimethoxy‐*E*‐citral	**9**	—	49
Fruits	3,7‐dimethyloctanoic acid	**10**	—	49
Fruits	neryl acetate	**11**	—	49
Fruits	geranyl acetate	**12**	—	49
Fruits	methyl geranate	**13**	—	49
Fruits	neryl acetone	**14**	—	49
Flowers	α‐phellandrene	**15**	—	5
Flowers	α‐pinene	**16**	Antinociceptive	5, 7
Flowers	camphene	**17**	Insecticidal	5, 8
Flowers	*m*‐cymene	**18**	—	5
Flowers	terpinolene	**19**	—	5
Flowers	terpinen‐4α‐ol	**20**	—	5
Leaves	verbanol acetate	**21**	—	6, 49
**Sesquiterpenes**				
Leaves and flowers	spathulenol	**22**	Antimicrobial and antifungal	5, 6, 12, 13, 43, 49
Leaves and flowers	germacrene‐D	**23**	Antitumoral, antimicrobial, and antifungal	6, 43, 49
Leaves	biciclogermacrene	**24**	Antitumoral, antimicrobial, and antifungal	6, 12, 13, 43, 49
Leaves	macrocarp‐11(15)‐en‐8‐ol	**25**	—	12, 13
Fruits	α‐ylangene	**26**	—	49
Fruits	β‐bourbonene	**27**	—	49
Fruits	*E*‐caryophyllene	**28**	—	49
Fruits	β‐duprezianene	**29**	—	49
Fruits	*E*‐β‐farnesane	**30**	—	49
Leaves and fruits	γ‐muurolene	**31**	—	43, 49
Fruits	α‐amorphene	**32**	—	49
Fruits	*cis*‐eudesma‐6,11‐diene	**33**	—	49
Fruits	α‐muurolene	**34**	—	49
Fruits	butylated hydroxytoluene	**35**	—	49
Leaves, flowers, and fruits	δ‐cadinene	**36**	Antibacterial	5, 9, 13, 43, 49
Leaves and fruits	*trans*‐cadina‐1,4‐diene	**37**	—	43, 49
Fruits	α‐cadinene	**38**	—	49
Leaves and fruits	α‐calacorene	**39**	—	43, 49
Flowers and fruits	*E*‐nerolidol	**40**	—	5, 49
Fruits	dendrolasin	**41**	—	49
Leaves and fruits	globulol	**42**	—	13, 43, 49
Fruits	viridiflorol	**43**	—	49
Fruits	cubebol	**44**	—	49
Leaves and fruits	geranyl 2‐methylbutyrate	**45**	—	43, 49
Fruits	geranyl isovalerate	**46**	—	49
Fruits	5‐*epi*‐7‐*epi*‐α‐eudesmol	**47**	—	49
Fruits	himachalol	**48**	—	49
Fruits	α‐cadinol	**49**	—	49
Leaves and fruits	*E*‐bisabol‐11‐ol	**50**	—	6, 49
Fruits	(*Z*)‐nerolidyl acetate	**51**	—	49
Fruits	α‐bisabolol	**52**	—	49
Fruits	davanol acetate	**53**	—	49
Fruits	2*E*,6*E*‐farnesol	**54**	—	49
Fruits	β‐bisabolenal	**55**	—	49
Fruits	5*E*,9*E*‐farnesyl acetone	**56**	—	49
Fruits	α‐gurjunene	**57**	—	5
Flowers	allo‐aromadendrene	**58**	—	5
Flowers	humulene	**59**	Anticancer and insecticidal	5, 8, 10
Flowers	germacrene‐B	**60**	—	5, 43
Leaves and flowers	δ‐elemene	**61**	—	43
Leaves	bicycloelemene	**62**	Antimicrobial and antifungal	22, 43
Leaves	α‐cubebene	**63**	—	13, 43
Leaves	α‐copaene	**64**	—	6, 13, 43, 49
Leaves	daucene	**65**	—	43
Leaves	β‐cubebene	**66**	—	43
Leaves	β‐elemene	**67**	Antitumoral	10, 43
Leaves	β‐cedrene	**68**	—	6, 43, 49
Leaves	β‐copaene	**69**	—	43
Leaves	aromadendrene	**70**	—	43
Leaves	amorpha‐4,7(11)‐diene	**71**	—	43
Leaves	*cis*‐β‐guaiene	**72**	—	43
Leaves	γ‐cadinene	**73**	—	13, 43
Leaves	rosifoliol	**74**	—	43
Leaves	guaiol	**75**	—	43
Leaves	β‐atlantol	**76**	—	43
Leaves	isolongifolan‐7α‐ol	**77**	—	43
Leaves	junenol	**78**	—	43
Leaves	*trans*‐isolongifolanone	**79**	—	43
Leaves	1‐*epi*‐cubenol	**80**	—	13, 43
Leaves	α‐muurolol	**81**	—	43
Leaves	cubenol	**82**	—	43
Leaves	valerianol	**83**	—	43
Leaves	7‐*epi*‐α‐eudesmol	**84**	—	43
Leaves	mustacone	**85**	—	43
Leaves	eudesma‐4(15),7‐dien‐1β‐ol	**86**	—	43
Leaves	germacrone	**87**	—	43
Leaves	cyperotundone	**88**	Anticancer	11, 43
Leaves	amorpha‐4,9‐dien‐2‐ol	**89**	—	43
Leaves	amorpha‐4,9‐dien‐14‐al	**90**		43
Leaves	2β‐hydroxyvalencene	**91**	—	43
Leaves	α‐guaiene	**92**	—	6, 49
Leaves	isolonfigolene	**93**	—	6, 49
**Diterpenes**				
Flowers	*E*‐phytol	**94**	P‐gp inhibitor	12, 13, 14, 49
Fruits	isophytol	**95**	—	49
Fruits	3*Z*‐cembrane A	**96**	—	49
Fruits	*E*,*E*‐geranyl linalool	**97**	—	49
Fruits	manool	**98**	—	49
Fruits	*E*‐phytol acetate	**99**	—	49
**Hemiterpenes**				
Branches/twigs	3‐methylenebutane‐1,2,4‐triol	**100**	—	12, 15
**Alkaloids**				
Branches/twigs	reticuline	**101**	antimicrobial and anticancer	12, 15, 16, 18, 19
Branches/twigs	4’‐methylcoclaurine	**102**	—	12, 16
Branches/twigs	coclaurine	**103**	Cardiovascular effect, antispasmodic, anti‐HIV, and anticancer	12, 16, 22, 23, 24, 50
Branches/twigs	norjuziphine	**104**	Anti‐platelet aggregation	21, 12, 16
Branches/twigs	asimilobine	**105**	Antioxidant, antimicrobial, and anticancer	12, 16, 18, 19
Branches/twigs	michelalbine	**106**	—	12, 16
Branches/twigs	stefarine	**107**	—	12, 16
Branches/twigs	liriodenine	**108**	Antimicrobial, anticancer, and anti‐COVID	12, 16, 18, 19, 25
Leaves and branches/twigs	cleistopholine	**109**	Antimicrobial, antifungal, and anticancer	12, 16, 17, 18, 19
Leaves and branches/twigs	6‐methoxycleistopholine	**110**	Antifungal	12, 16, 17
Branches/twigs	6,7‐dimethoxycleistopholine	**111**	—	12, 16
Branches/twigs	5‐hydroxy‐6‐methoxycleistopholine	**112**	—	12, 16
Branches/twigs	5‐hydroxy‐6,7‐dimethoxycleistopholine	**113**	Antifungal	12, 16, 17
Branches/twigs	onichine	**114**	Antifungal	12, 16, 17
Branches/twigs	6‐methoxyonichine	**115**	Antifungal	12, 16, 17
Branches/twigs	6,7‐ dimethoxyonichine	**116**	Antifungal	12, 16, 17
Branches/twigs	7‐methoxyonichine	**117**	Antifungal	12, 16, 17
Flowers	2‐methoxy‐3‐(1‐methylpropyl)‐pyrazine	**118**	—	5
**Amino acids**				
Branches/twigs	18‐citrulline	**119**	—	12, 49
Branches/twigs	19‐pipecolic acid	**120**	—	12, 49
Branches/twigs	*L*‐valine	**121**	—	12, 49
Branches/twigs	*L*‐proline	**122**	—	12, 49
Branches/twigs	*L*‐alanine	**123**	—	12, 49
Branches/twigs	γ‐aminobutyric acid	**124**	—	12, 49
Branches/twigs	4‐hydroxyproline	**125**	—	12, 49
**Arylpropanoids**				
Branches/twigs	*N*‐*trans*‐feruloyltyramine	**126**	Antidiabetic, anti‐inflammatory, anticancer, antibacterial, antifungal, and antioxidant	12, 15, 27, 28, 29, 31, 32, 33, 52
Branches/twigs	*N*‐*trans*‐cafeoyltyramine	**127**	Antifungal, antibacterial, and anticancer	12, 15, 31, 32, 52
Branches/twigs	*N*‐*trans*‐sinapoylyiramine	**128**	Anticancer	12, 15, 33, 52
Branches/twigs	lignanamide A	**129**	—	12, 15, 52
Branches/twigs	lignanamide B	**130**	—	12, 15, 52
**Steroids**				
Leaves and branches/twigs	stimast‐4‐en‐3‐one (sitostenone)	**131**	—	12, 35
Leaves and branches/twigs	3β‐stigmasta‐4,22,25‐trien‐3‐ol	**132**	—	12, 35
Branches/twigs	stigmasta‐4,25‐dien‐3‐one	**133**	—	12, 35
Branches/twigs	stigmasta‐4,22*E*,25‐trien‐3‐one	**134**	—	12, 35
Branches/twigs	3‐*O*‐β‐glucosyl‐sistosta‐5,25‐dien‐3‐ol	**135**	—	12, 35
Branches/twigs	3‐*O*‐β‐glucosyl‐sistosta‐5,22,25‐trien‐3‐ol	**136**	—	12, 35
**Flavonoids**				
Leaves and branches/twigs	3‐O‐rutinosyl‐7,4'‐O‐dimethyl quercetin	**137**	—	12, 35
Leaves and branches/twigs	3‐O‐rutinosyl‐5‐β‐O‐glucopyranosyl‐7,4'‐O‐dimethyl quercetin	**138**	—	12, 15, 35
**Hydrocarbons**				
Fruits	1‐octadecene	**139**	—	49
Fruits	pentacosane	**140**	—	49
Fruits	hexacosane	**141**	—	49
Fruits	heptacosane	**142**	P‐gp inhibitor	14, 49
Flowers	toluene	**143**	—	5
**ORGANIC SALTS**				
Branches/twigs	tyrosine trimethylammonium chloride	**144**	—	12, 15
Branches/twigs	glycine trimethylammonium chloride	**145**	—	12, 15
Leaves	choline chloride	**146**	—	12, 15
**Acylglycerols**				
Seeds	α,α’‐dimacrocarpoyl‐β‐oleylglycerol	**147**	—	36
Seeds	α‐macrocarpoyl‐α’‐oleylglycerol	**148**	—	36
**Fatty acids**				
Seeds	eicos‐11‐yn‐19‐enoic acid	**149**	—	12
Seeds	12,14‐octadecadiynoic acid (macrocarpic acid)	**150**	Anti‐*T. cruzi*	49, 36
Flowers	octadec‐11‐en‐9‐ynoic acid (santalbic acid)	**151**	Anti‐*T. cruzi*	5, 37
Flowers	8‐hydroxyoctadeca‐9,11‐diynoic acid	**152**	Anti‐*T. cruzi*	5, 37
Flowers	8‐hydroxyoctadec‐17‐ene‐9,11‐diynoic acid	**153**	—	5
Flowers	octadec‐9‐ynoic acid (stearolic acid)	**154**	Anti‐*T. cruzi*	5, 37
Seeds	docos‐13‐yn‐21‐enoic acid	**155**	Antileishmanial	38
**Lactone**				
Fruits	γ‐dodelactone	**156**	—	49
**Acetogenins**				
Seeds	(2*S*,3*R*,4*R*)‐3‐hydroxy‐4‐methyl‐2‐(*n*‐eicos‐11’‐yn‐19’‐enyl)butanolide	**157**	Antitumoral, anti‐*T. cruzi* and antileishmanial	12, 38, 39, 52, 53
Seeds	(2*S*,3*R*,4*R*)‐3‐hydroxy‐4‐methyl‐2‐(*n*‐eicos‐11’‐ynyl)butanolide	**158**	Antitumoral	12, 39, 52, 53
Seeds	3‐hydroxy‐4‐methylen‐2‐(*n*‐eicos‐11′‐yn‐19′‐enyl)but‐2‐enolide	**159**	Anti‐*T. cruzi* and antileishmanial	38, 39
Seeds	(4*R*)‐3‐hydroxy‐4‐methyl‐2‐(*n*‐eicos‐11′‐yn‐19′‐enyl)but‐2‐enolide	**160**	Anti‐*T. cruzi*	39
Seeds	3‐hydroxy‐4‐metilen‐2‐(octadeca‐9’‐yn‐17’‐enyl)but‐2‐enolide	**161**	Antileishmanial	38
Seeds	3‐hydroxy‐4‐methylen‐2‐(*n‐*hexadeca‐7’‐yn‐15’‐enyl)but‐2‐enolide	**162**	Antileishmanial	38
Seeds	(+)‐(2*S*,3*R*,4*R*)‐3‐hydroxy‐4‐methyl‐2‐(*n*‐octadec‐11′‐yn‐17′‐enyl)butanolide	**163**	Anti‐*T. cruzi*	41
Seeds	(+)‐(2*S*,3*R*,4*R*)‐3‐hydroxy‐4‐methyl‐2‐(*n*‐docos‐11′‐yn‐21′‐enyl)butanolide	**164**	Anti‐*T. cruzi*	41
Seeds	(+)‐(2*S*,3*R*,4*R*)‐3‐hydroxy‐4‐methyl‐2‐(*n*‐tetracos‐11′‐yn‐23′‐enyl)butanolide	**165**	Anti‐*T. cruzi*	41
Seeds	(+)‐(2*S*,3*R*,4*R*)‐3‐hydroxy‐4‐methyl‐2‐(*n*‐octadec‐11′‐ynyl)butanolide	**166**	Anti‐*T. cruzi*	41
Seeds	(+)‐(2*S*,3*R*,4*R*)‐3‐hydroxy‐4‐methyl‐2‐(*n*‐docos‐11′‐ynyl)butanolide	**167**	Anti‐*T. cruzi*	41
Seeds	(+)‐(2*S*,3*R*,4*R*)‐3‐hydroxy‐4‐methyl‐2‐(*n*‐tetracos‐11′‐ynyl)butanolide	**168**	Anti‐*T. cruzi*	41
Fruits	(2*S*,3*R*,4*R*)‐3‐hydroxy4‐methyl‐2‐(*n*‐octadeca‐13′,17′‐dien‐11′‐ynyl)butanolide	**169**	Anti‐*T. cruzi*	42
Fruits	(2*S*,3*R*,4*R*)‐3‐hydroxy‐4‐methyl‐2‐(*n*‐eicosa‐13′,19′‐dien‐11′‐ynyl)butanolide	**170**	Anti‐*T. cruzi*	42
Fruits	(2*S*,3*R*,4*R*)‐3‐hydroxy‐4‐methyl‐2‐(*n*‐octadec‐13′‐en11′‐ynyl)butanolide	**171**	Anti‐*T. cruzi*	42
Fruits	(2*S*,3*R*,4*R*)‐3‐hydroxy‐4‐methyl‐2‐(*n*‐eicosa‐13′‐en‐11′‐ynyl)butanolide	**172**	Anti‐*T. cruzi*	42
**OTHER COMPOUNDS**				
Leaves	2‐hydroxy‐1,4‐dimethoxy‐3‐methyl‐9*H*‐fluoren‐9‐one	**173**	—	12
Branches/twigs	1,3,5‐trimethylbenzene	**174**	—	12
Branches/twigs	glycerol	**175**	—	12
Fruits	phenylacetaldehyde	**176**	Antityrosinase and antimicrobial	44, 49
Fruits	methyl salicylate	**177**	Acaricide	45, 49
Fruits	oct‐2*E*‐en‐1‐ol	**178**	—	49
Fruits	dec‐2*E*‐enal	**180**	—	49
Fruits	*Z*‐α‐damascone	**181**	—	49
Fruits	*E*‐α‐damascone	**182**	—	49
Fruits	*n*‐hexadecanol	**183**	—	49
Fruits	ethyl hexadecanoate	**184**	—	49
Fruits	*n*‐octadecanol	**185**	—	49
Flowers	ethyl pentanoate	**186**	Anti‐quorum	5, 46
Flowers	isobutyl acetate	**187**	—	5
Flowers	1,1‐diethoxypropane	**188**	—	5
Leaves	2‐penthyl furan	**189**	—	43
Leaves	*n*‐nonanal	**190**	—	43
Leaves	2*Z*–nonen‐1‐al	**191**	—	43
Leaves	α‐amylcinnamyl alcohol	**192**	—	43
Branches/twigs	liriodendrin	**193**	Anticancer, anti‐asthmatic, anti‐inflammatory, and antinociceptive	12, 15, 47, 48, 54

## 3 CONCLUSIONS

Chemical studies of *P. macrocarpa* have revealed a diverse array of secondary metabolites, notably terpenoids, acetogenins, and alkaloids. Many of these compounds exhibit a wide range of biological activities—including antibacterial, antifungal, antiviral, cytotoxic, anti‐inflammatory, antiparasitic, and antioxidant effects—linking the plant's biological potency to its rich metabolite profile. Consequently, metabolites from *P. macrocarpa* represent a valuable resource for drug discovery, particularly in the development of new therapeutic prototypes targeting diseases that currently lack effective or widely accessible treatments.

## Author Contributions

**Fernanda Thevenard**: organized the materials, conceived the framework, analyzed the data, and wrote the article. **Ivanildo A. Brito**: organized the materials, conceived the framework, analyzed the data, and wrote the article. **Emerson A. Oliveira**: revised and proofread the final version of the manuscript. **Mariana H. Chaves**: revised and proofread the final version of the manuscript. **João Henrique G. Lago**: revised and proofread the final version of the manuscript.

## Funding

The authors would like to thank CNPq, FAPESP (2023/12447‐1 and 2020/01221‐4), and CAPES (Finance Code 001). We are also thankful to the CNPq scientific research grant awarded to João Henrique G. Lago and Mariana H. Chaves.

## Conflicts of Interest

The authors declare no conflicts of interest.

## Data Availability

The data that support the findings of this study are available from the corresponding author upon reasonable request.
